# LncRNA HOTAIRM1 promotes MDSC expansion and suppressive functions through the HOXA1-miR124 axis during HCV infection

**DOI:** 10.1038/s41598-020-78786-1

**Published:** 2020-12-16

**Authors:** Bal Krishna Chand Thakuri, Jinyu Zhang, Juan Zhao, Lam N. Nguyen, Lam N. T. Nguyen, Sushant Khanal, Dechao Cao, Xindi Dang, Madison Schank, Xiao Y. Wu, Zheng D. Morrison, Mohamed El Gazzar, Zhengke Li, Yong Jiang, Shunbin Ning, Ling Wang, Jonathan P. Moorman, Zhi Q. Yao

**Affiliations:** 1 Center of Excellence for Inflammation, Infectious Disease and Immunity, James H. Quillen College of Medicine, East Tennessee State University, Johnson City, TN 37614 USA; 2 Division of Infectious, Inflammatory and Immunologic Diseases, Department of Internal Medicine, Quillen College of Medicine, ETSU, Johnson City, TN 37614 USA; 3 Department of Veterans Affairs, Hepatitis (HCV/HBV/HIV) Program, James H. Quillen VA Medical Center, Johnson City, TN 37614 USA; 4 Center of Excellence for HIV/AIDS Care, Quillen College of Medicine, East Tennessee State University, Johnson City, TN 37614 USA

**Keywords:** Immunology, Diseases, Medical research

## Abstract

HOXA transcript antisense RNA myeloid-specific 1 (HOTAIRM1) is a long non-coding RNA (lncRNA) that plays a pivotal role in regulating myeloid cell development via targeting HOXA1 gene expression. We and others have previously shown that myeloid-derived suppressor cells (MDSCs), a heterogeneous population of immature myeloid cells, expand during chronic viral (HCV, HIV) infections. However, the role of HOTAIRM1 in the development and suppression of MDSCs during viral infection remains unknown. In this study, we demonstrate that the expressions of HOTAIRM1 and its target HOXA1 are substantially upregulated to promote the expressions of immunosuppressive molecules, including arginase 1, inducible nitric oxide synthase, signal transducer and activator of transcription 3, and reactive oxygen species, in CD33^+^ myeloid cells derived from hepatitis C virus (HCV)-infected patients. We show that HCV-associated exosomes (HCV-Exo) can modulate HOTAIRM1, HOXA1, and miR124 expressions to regulate MDSC development. Importantly, overexpression of HOTAIRM1 or HOXA1 in healthy CD33^+^ myeloid cells promoted the MDSC differentiation and suppressive functions; conversely, silencing of HOTAIRM1 or HOXA1 expression in MDSCs from HCV patients significantly reduced the MDSC frequency and their suppressive functions. In essence, these results indicate that the HOTAIRM1-HOXA1-miR124 axis enhances the differentiation and suppressive functions of MDSCs and may be a potential target for immunomodulation in conjunction with antiviral therapy during chronic viral infection.

## Introduction

Hepatitis C virus (HCV) is characterized by a high rate (> 80%) of chronic infection^1,2^ and thus it has become an excellent model for studying the mechanisms of virus-induced host immune dysfunction and viral chronicity in humans. While the use of direct acting antiviral (DAA) agents can efficiently clear HCV infection, these therapeutic drugs faces new issues such as viral mutation, relapse, and reinfection following treatment^3,4^. According to the CDC report, the number of HCV-related deaths has reached an all-time high, surpassing 60 other nationally reportable infectious diseases combined, making hepatitis C the number one reportable infectious disease that kills people in the United States^5^. Therefore, further studying the mechanisms that can dampen host immunity to permit viral persistence is essential to better managing chronic viral infections.

Myeloid-derived suppressor cells (MDSCs) are a heterogeneous population of immature myeloid cells generated due to aberrant myelopoiesis under various pathological conditions, such as cancer, inflammatory and infectious diseases^6–8^. While MDSCs may contribute to immune homeostasis via limiting excessive inflammatory processes, their expansion may be at the expense of pathogen elimination, and thus they promote persistent infection^8^. We and others have previously reported that MDSCs expansion can inhibit T cell function in multiple disease models, including chronic HCV and HIV infections^9–16^. However, the mechanisms that drive MDSCs differentiation and suppressive functions during viral infection remain unclear.

Long non-coding RNAs (lncRNAs) are genomic transcripts > 200-nt in length that do not encode proteins but possess regulatory functions^17–19^. By using RNA sequencing and annotation of the GENECODE project^20^, thousands of lncRNAs have been discovered recently, but their functions have not been well-characterized. Of note, the expression of lncRNAs is species-, cell-, and disease stage-specific^17–19^. The HOXA gene cluster, which is specifically expressed in the myeloid lineage, has been shown to generate lncRNAs with transcriptional regulatory functions in myelopoiesis^21–23^. Mechanistically, a class of transcripts in the HOXA region, termed HOTAIR, act in *trans* to control target gene expression remotely by recruiting histone methyltransferases^21^, whereas other HOXA intergenic transcripts act in *cis* to control neighboring HOXA genes^22,23^. Particularly, HOXA transcript antisense RNA myeloid-specific 1 (HOTAIRM1) is an intergenic lncRNA encoded in the HOXA gene cluster, and appears to be the most prominent lncRNA upregulated during granulocyte differentiation and myeloid cell maturation^24–26^. While the regulatory effects of HOTAIRM1-HOXA1 axis on hematopoiesis, leukemogenesis, and oncogenesis have been reported^27–31^, the potential roles of HOTAIRM1 in controlling viral infections, especially via regulation of MDSCs differentiation and function during HCV infection, remain largely unknown.

In this study, we characterized the expression and function of lncRNAs in MDSCs development. We found that HOTAIRM1 is upregulated during HCV infection and drives MDSCs expansion through regulating HOXA1 and miR124 expressions. We also found that HCV-containing exosomes (HCV-Exo) dysregulate the HOTAIRM1-HOXA1-miR124 axis, playing an important role in regulating the immunosuppressive functions of MDSCs. Our study reveals a novel mechanism of immune dysregulation during chronic viral infection.

## Materials and methods

### Subjects

The study protocol was approved by the joint Institutional Review Board (IRB) of East Tennessee State University and James H. Quillen VA Medical Center (ETSU/VA IRB, Johnson City, TN). Written informed consent was obtained from all participants. All methods were performed in accordance with the relevant guidelines and regulations. The study subjects were composed of two populations: 50 chronically HCV-infected individuals and 54 healthy subjects (HS). HCV genotype (70% type 1, 30% type 2 or 3) and viral load (ranging from 17,000 ~ 17,000,000 IU/ml) were performed by Lexington VAMC, and all subjects were virologically and serologically positive for HCV prior to antiviral treatment. Healthy subjects were negative for HBV, HCV, and HIV infections, and blood buffy coats were obtained from Key Biologics (Memphis, TN) or Physician Plasma Alliance LLC (Gray, TN).

### Cell isolation, culture, and flow cytometric analysis

Peripheral blood mononuclear cells (PBMCs) were isolated from whole blood using Ficoll density gradients (GE Healthcare, Piscataway, NJ). CD33^+^ cells were isolated from PBMCs using a CD33^+^ Cell Isolation Kit and a MidiMACS Separator column (Miltenyi Biotec Inc., Auburn, CA). The cells were cultured in RPMI 1640 medium with 10% fetal bovine serum (Atlanta Biologicals, Flowery Branch, GA), 100 IU/ml penicillin, and 2 mM l-glutamine (Thermo Scientific, Logan, Utah) at 37 °C and 5% CO_2_ atmosphere. Cell depletion and cell culture with exosomes were carried out as described previously^13–16^. Flow cytometry analysis of cell phenotypes and intracellular cytokines in PBMCs was carried out as described previously^16^. The following reagents were used: anti-CD4-FITC (Biolegend), anti-IFN-γ-PE (Biolegend), anti-CD33-PE(Biolegend), anti-CD14-APC (Biolegend), anti-HLA-DR-FITC (Biolegend), anti-CD3-APC (Biolegend), anti-Arg1-PE (Biolegend), anti-pSTAT3-PerCP (Biolegend) and anti-iNOS-PE (Novus biologicals) along with isotype control antibodies (BD Bioscience, San Jose, CA). Levels of reactive oxygen species (ROS) in myeloid cells were measured using the H2DCFDA-based kit (Invitrogen) according to manufacturer’s protocol. The stained cells were acquired on an Accuri C6 flow cytometer (BD, Franklin Lakes, NJ) and analyzed using FlowJo software (Tree Star, Inc., Ashland, OR). Isotype control antibodies (eBioscience) and fluorescence minus one (FMO) controls were used to determine the background levels of staining and adjust multicolor compensation as gating strategy.

### Exosome isolation and purification

Plasma was purified from 50 ml of whole blood from the research subjects and filtered to exclude particles larger than 0.8 μm, using syringe filters (Millipore Millex-AA Cat. No: SLAA033SS, Billerica, MA). Exosomes were then isolated from plasma by a differential centrifugation method as previously described^15,16^.

### lncRNA, miRNA arrays, and RT-PCR validation

CD33^+^ myeloid cells were purified from PBMCs as described above. Total cellular RNA from CD33^+^ cells was isolated using the miRNeasy Mini kit (Qiagen, Valencia, CA). The RNA quality and quantity were analyzed using a BioPhotometer spectrophotometer UV/VIS, and RNA integrity was determined using gel electrophoresis. lncRNAs were analyzed using the Arraystar gene array service. The miScript miRNA array was performed by Qiagen Inc. (Valencia, CA)^15^. To validate the results for up- or down-regulated miRNAs by real-time PCR, cDNA was generated from total RNA by the Taqman advanced miRNA cDNA synthesis kit and the High-Capacity cDNA Reverse Transcription Kit (Thermo Scientific, Logan, Utah). The miRNA expression levels were assessed by RT-PCR using Taqman fast advanced master mix (Thermo Scientific) and the CFX96 RT-PCR Detection System (Bio-Rad Laboratories Inc, Hercules, CA). The miRNA levels were determined using the 2^−ΔΔct^ relative quantification method and were normalized to internal control miR-191 or U6 RNA (SNORD61).

### Transfection and co-culture experiments

For miR124 inhibition, CD33^+^ cells isolated from PBMCs of HS were transfected with 30 pmol of miR124 inhibitor, or negative control. For HOTAIRM1 and HOXA1 silencing, the cells were transfected with 50 nM HOTAIRM1 SMART pool siRNA, HOXA1 SMART pool siRNA, or a pool of scrambled siRNA (Lafayette, CO, USA). For HOTAIRM1 and HOXA1 overexpression, the CD33^+^ cells were transfected with HOTAIRM1 or HOXA1 expression plasmid containing GFP (GenScript). Transfection efficiency was checked by flow cytometry 2 days after transfection. The transfection of miRNA or siRNA was performed using the Human Monocyte Nucleofector Kit and Nucleofector II Device (Lonza, Allendale, NJ) following the manufacturer’s instructions. The transfected cells were cultured for 2 days in IMEM medium (Lonza, Allendale, NJ) with 10% FBS. The cells were analyzed by flow cytometry or RT-PCR. For CD4^+^ T cell co-culture, autologous CD4^+^ T cells were stimulated with anti-CD3 and anti-CD28 (1 μg/ml each; BD Bioscience) for 2 days in IMEM complete medium, followed by adding the transfected CD33^+^ cells (at 1:2 ratio) for another two days.

### Statistical analysis

The parametric data are presented as mean ± SEM. Comparison between two groups was analyzed using unpaired t test with Welch’s correction after checking the value of F test. One-tail paired *t*-test was used to compare two groups. The nonparametric data (Fig. 3F) are presented as median with interquartile range and were analyzed by a one-tail Mann Whitney test. *P*-values < 0.05 or *P* < 0.01 were considered significant or very significant, respectively.

## Results

### MDSCs expand during chronic HCV infection

**Figure 1:**
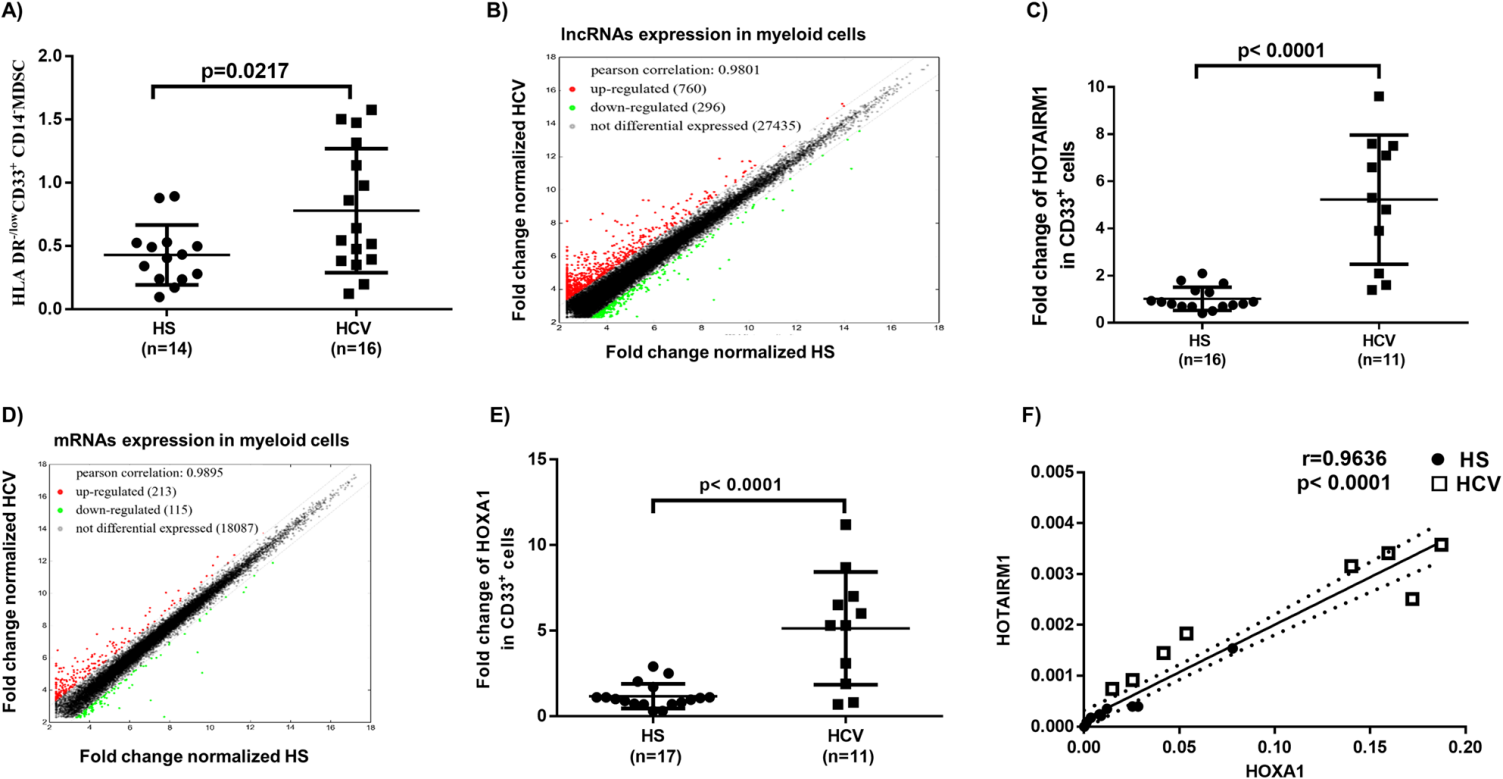
HOTAIRM1 and HOXA1 are upregulated in MDSCs in HCV-infected individuals. (**A**) Expansion of G-MDSCs (HLA-DR^−/low^CD33^+^CD14^−^ cells) in PBMCs of HCV-infected individuals compared with HS, as determined by flow cytometry. (**B**) Scatter plot of the heat map of lncRNA expression in CD33^+^ cells isolated from HCV-infected individuals versus HS (n = 5 per group). (**C)** HOTAIRM1 expression in CD33^+^ cells isolated from HCV-infected individuals versus HS, as determined by real-time RT-PCR. (**D**) Scatter plot of the heat map of mRNA expression in CD33^+^ cells isolated from HCV-infected individuals versus HS (n = 5 per group). (**E**) HOXA1 expression in CD33^+^ cells isolated from HCV-infected individuals versus HS, as determined by RT-PCR. (**F**) Pearson Correlation analysis of HOTAIRM1 and HOXA1 expression levels in CD33^+^ cells derived from the same subjects.

MDSCs play a critical role in disease progression by suppressing host immune responses^6–8,32^. The phenotypic marker of immature myeloid cells (CD33^+^ HLA-DR^−/low^) is a characteristic feature of human suppressive MDSCs, which are further categorized into monocytic MDSCs (M-MDSCs) and granulocytic MDSCs (G-MDSCs) based on the differential expression of the CD14 and CD15 markers, respectively^33,34^. We have recently reported an expansion of M-MDSCs, which inhibited T cell functions via promoting regulatory T cell (Treg) differentiation during chronic viral infections^13–16^. To better understand the role of MDSCs in chronic HCV infection, we further analyzed the frequencies of MDSCs in peripheral blood mononuclear cells (PBMCs) isolated from HCV patients and HS controls by flow cytometry. We found that the frequencies of G-MDSCs (CD33^+^ HLA-DR^−/low^ CD14^−^) were also increased in PBMCs in individuals with chronic HCV infection when compared to healthy subjects (HS) (Fig. 1A).

### HOTAIRM1 and HOXA1 are upregulated in MDSCs during chronic HCV infection

To determine whether lncRNAs play any role in MDSC expansion during HCV infection, we analyzed the transcripts of lncRNAs and messenger RNAs (mRNAs) in myeloid cells isolated from HCV-infected individuals and HS using the Arraystar gene array analysis. Among the lncRNAs analyzed (shown as scatter plot in Fig. 1B), 760 lncRNAs (red dots) were upregulated (> twofold), 296 lncRNAs (green dots) were downregulated, and 27,435 lncRNAs (black dots) remain unchanged in myeloid cells from HCV subjects compared to HS. Given the critical role of HOTAIRM1 in granulocyte differentiation and myeloid cell maturation^24–26^, we analyzed the HOTAIRM1 expression in gene array results and validated the results by RT-PCR, which revealed a fivefold increase in myeloid cells derived from HCV-infected individuals (Fig. 1C).

Among the mRNA analyzed (shown as scatter plot in Fig. 1D), 213 mRNAs (red dots) were upregulated, 115 mRNAs (green dots) were downregulated, and 18,087 mRNA transcripts remained unchanged (within a twofold limit). Notably, the mRNA array analysis showed a significant upregulation of HOXA1 (the HOTAIRM1 target gene) in CD33^+^ cells derived from HCV subjects and the results were validated by RT-PCR that showed a fivefold increase in HCV subjects (Fig. 1E). Importantly, the levels of HOTAIRM1 were positively correlated with HOXA1 expression according to Pearson Correlation analysis (Fig. 1F). Taken together, these results suggest that expressions of HOTAIRM1 and its target gene HOXA1 are concurrently upregulated and may serve as a biomarker for MDSC expansion during HCV infection.

### Immunosuppressive molecules are elevated in MDSCs during chronic HCV infection

**Figure 2:**
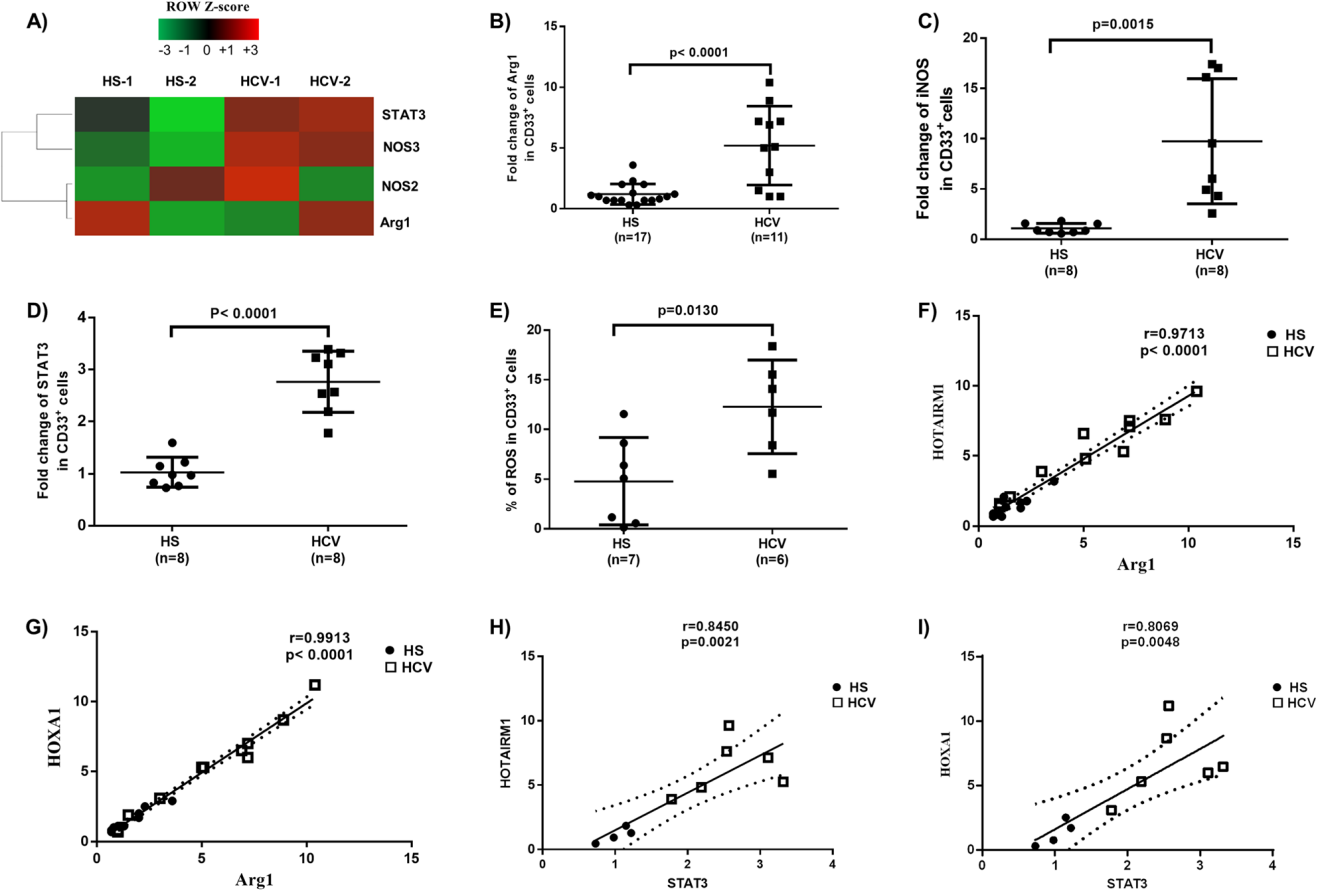
Upregulation of immunosuppressive molecules in MDSCs during chronic HCV infection. (**A**) Levels of STAT3, NOS3, NOS2, and Arg1 mRNAs in CD33^+^ cells isolated from HCV-infected individuals versus HS, analyzed by gene array. (**B–D**) Levels of Arg1, iNOS, and STAT3 gene expressions in CD33^+^ cells isolated from HCV-infected individuals versus HS, analyzed by RT-PCR. (**E**) ROS production in CD33^+^ cells derived from HCV-infected individuals versus HS, analyzed by the H2DCFDA assay. (**F**–**I**) Relationship between HOTAIRM1 or HOXA1 and Arg1 or STAT3 expression levels, analyzed by Pearson Correlation analysis.

MDSCs suppress immune responses by producing immunosuppressive mediators, such as arginase 1 (Arg1), inducible nitric oxide synthase (iNOS), signal transducer and activator of transcription 3 (STAT3), and reactive oxygen species (ROS). Arg1 is constitutively expressed in granulocytes and represents a novel antimicrobial effector through arginine depletion in the phagolysosome^35^. iNOS catalyzes the production of superoxide and free radical nitric oxide as an immune regulator^36^. STAT3 is a transcription factor and plays a pivotal role in MDSC differentiation and suppressive functions^13–16^. ROS activate anti-oxidative pathways and induce transcriptional programs that regulate the differentiation and function of MDSCs as a part of a major mechanism to suppress T cell responses^36^. To determine the mechanisms by which MDSCs exert their immunosuppressive effects during HCV infection, we measured the mRNA levels of those molecules that are implicated in myeloid cell differentiation and functions. As shown in Fig. 2A, gene array analysis showed upregulation of STAT3, NOS3, NOS2, and Arg1 mRNA levels in CD33^+^ cells isolated from HCV patients. These findings were validated by RT-PCR, which revealed a 5 fold increase in Arg1 (Fig. 2B), a tenfold increase in iNOS (Fig. 2C), a 2.5-fold increase in STAT3 (Fig. 2D), and we also found a 2.6-fold increase in ROS production (Fig. 2E). Of note, the Arg1 and STAT3 levels positively correlated with HOTAIRM1 and HOXA1 expression levels in these subjects (Fig. 2F–I), suggesting the possibility that HCV-induced MDSCs may suppress immune responses by upregulating these immunosuppressive molecules through the HOTAIRM1-HOXA1 axis during HCV infection.

### miR124 expression negatively correlates with HOTAIRM1 levels in MDSCs during HCV infection

**Figure 3:**
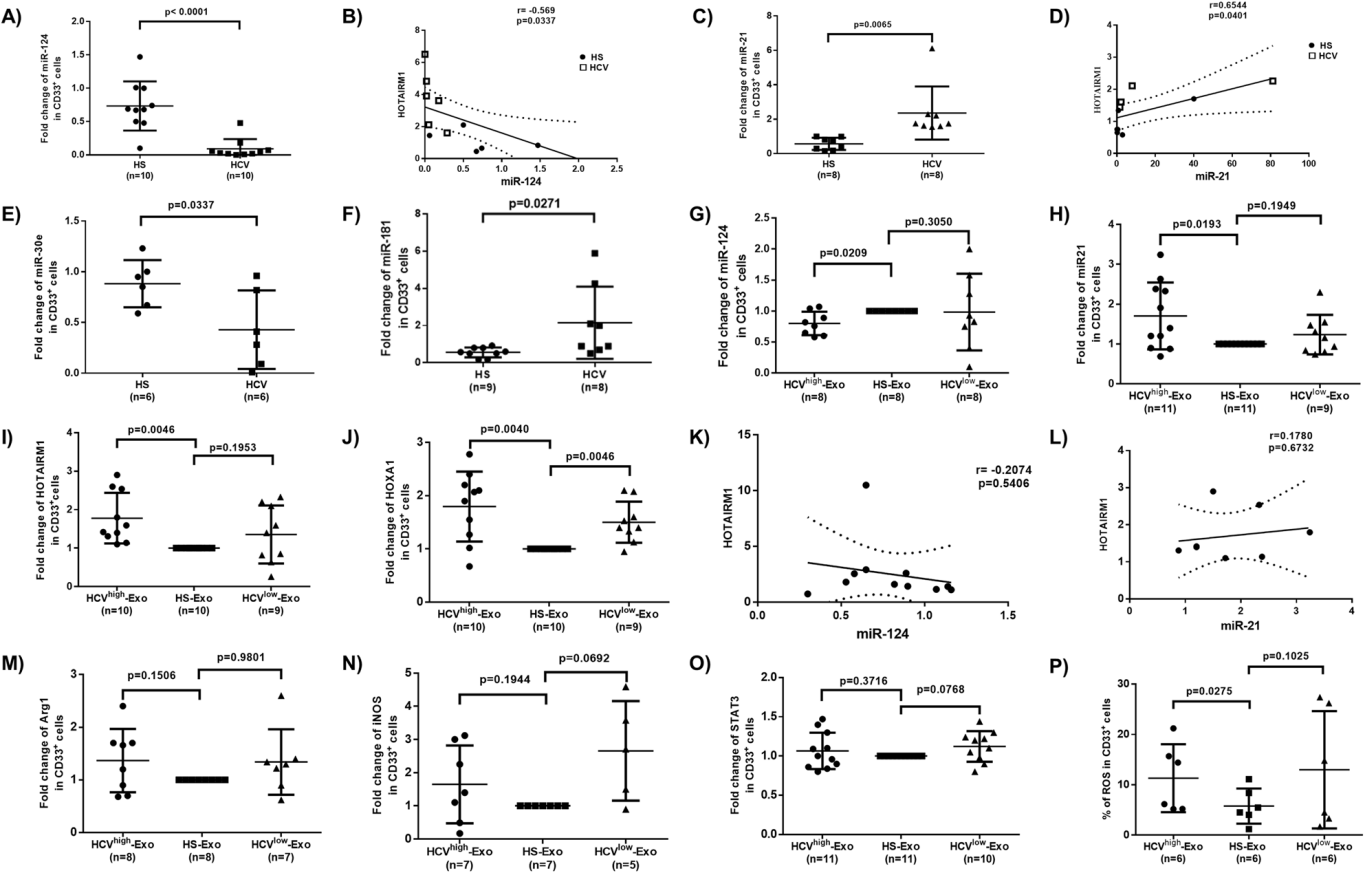
Analysis of miRNA, lncRNA, and mRNA expression levels in MDSCs during HCV infection in vivo and by HCV-Exo treatment in vitro. (**A–F**) Expression levels of miR124, miR21, miR30, miR181 and their correlation with HOTAIRM1 level in CD33^+^ cells isolated from HCV-infected individuals versus HS. (**G–L**) Expression levels of miR124, miR21, HOTAIRM1, HOXA1 and their correlations in CD33^+^ cells treated with exosomes isolated from plasma of HCV-infected individuals (HCV^high^-Exo or HCV^low^-Exo) and HS (HS-Exo). (**M–P**) Expression levels of Arg1, iNOS, STAT3, and ROS levels in CD33^+^ cells cultured with exosomes that were purified from plasma of HCV-infected individuals (HCV^high^-Exo or HCV^low^-Exo) versus HS (HS-Exo).

In addition to lncRNAs, miRNAs are also involved in myelopoiesis orchestrated by interdependent interactions between cytokine receptors and transcription factors^8,37–39^. We and others have previously shown that miRNAs dysregulate myelopoiesis to generate MDSCs^15,16,39–42^. To identify specific miRNAs that could affect myelopoiesis during HCV infection, we profiled miRNA expressions in CD33^+^ cells isolated from HCV patients and HS^15^. Expression of miRNAs that were significantly dysregulated per the miRNA array results was confirmed by RT-PCR. Amongst the dysregulated miRNAs, we found that miR124 was significantly inhibited and its level negatively correlated with HOTAIRM1 expression (Fig. 3A–B), whereas miR21 was significantly upregulated and positively correlated with HOTAIRM1 expression (Fig. 3C–D). Although miR30 was the most down-regulated miRNA, as determined by gene array analysis and RT-PCR, it did not correlate with the HOTAIRM1 expression (Fig. 3E). Also, miR181, which we have previously shown to be downregulated to promote T cell senescence through upregulation of DUSP6^43^, was significantly increased in CD33^+^ cells during HCV infection, but did not correlate with the HOTAIRM1 expression (Fig. 3F). Expression of miR155, a miRNA that we have shown to be upregulated in NK cells to control their functions through regulating the Tim-3 pathway^44^, was also upregulated in myeloid cells during HCV infection but did not correlate with the HOTAIRM1 expression (data not shown). These data suggest that miR124 expression is negatively correlated to the HOTAIRM1 levels in MDSCs during HCV infection.

### HCV-Exo regulates miR124 and HOTAIRM1-HOXA1 expressions in MDSCs during HCV infection

Exosomes are membrane-bound extracellular microvesicles and serve as carriers to transfer various signaling molecules (such as viral RNA, mRNA or ncRNA) among cells without direct cell-to-cell contact, thus playing an important role in regulating immune responses^45–48^. Notably, the human tetraspanin CD81, a receptor for the HCV E2 glycoprotein, is enriched in exosomes^49^. HCV genomic materials can be released from infected hepatocytes into peripheral blood in the form of circulating exosomes, and these molecules can exploit the fusogenic capabilities of the exosomes with other cells to transmit HCV-RNA and to dysregulate the immune responses, even in the presence of neutralizing antibodies^50–52^. We have recently shown that exosomes isolated from the plasma of HCV patients contain HCV-RNAs that promote MDSC expansion to inhibit T cell function^16^. To determine whether HCV-Exo can induce the molecular changes we observed in CD33^+^ cells, we isolated exosomes from the plasma of HCV subjects with high or low viral load (HCV RNA = 17,000,000, or 17,000, named as HCV^high^-Exo, or HCV^low^-Exo, respectively) and HS (HS-Exo). These exosomes were added to cultures of healthy PBMCs for 5 days, followed by the selection of CD33^+^ cells from the PBMCs. Similar to our observations in CD33^+^ cells isolated from HCV and HS, RT-PCR analysis showed that miR124 was downregulated (Fig. 3G), whereas miR21 was significantly upregulated (Fig. 3H) in healthy CD33^+^ cells treated with HCV^high^-Exo compared to HS-Exo treatment. Also, miR30 was downregulated, whereas miR181 and miR155 were upregulated, but their expressions did not correlate with the HOTAIRM1 levels (data not shown). Additionally, while both HCV^high^-Exo and HCV^low^-Exo increased HOTAIRM1 (Fig. 3I) and HOXA1 (Fig. 3J) expressions, only HCV^high^-Exo treatment led to a significant increase in HOTAIRM1 expression in CD33^+^ cells. Notably, among the HCV-Exo-induced alterations in miRNA and HOTAIRM1 expression, miR124 negatively correlated with HOTAIRM1 expression (Fig. 3K), whereas miR21 positively correlated with HOTAIRM1, though these correlations were not statistically significant (Fig. 3L). Moreover, HCV-Exo moderately upregulated the expression levels of Arg1 (Fig. 3M), iNOS (Fig. 3N), STAT3 (Fig. 3O), and ROS production (Fig. 3P), however, these changes were also not statistically significant. These results suggest that HCV-Exo plays a role in differential regulation of miR124, miR21, HOTAIRM1, and HOXA1 expressions during HCV infection.

### HOTAIRM1 and HOXA1 regulate each other to control miR124 expression in MDSCs

**Figure 4:**
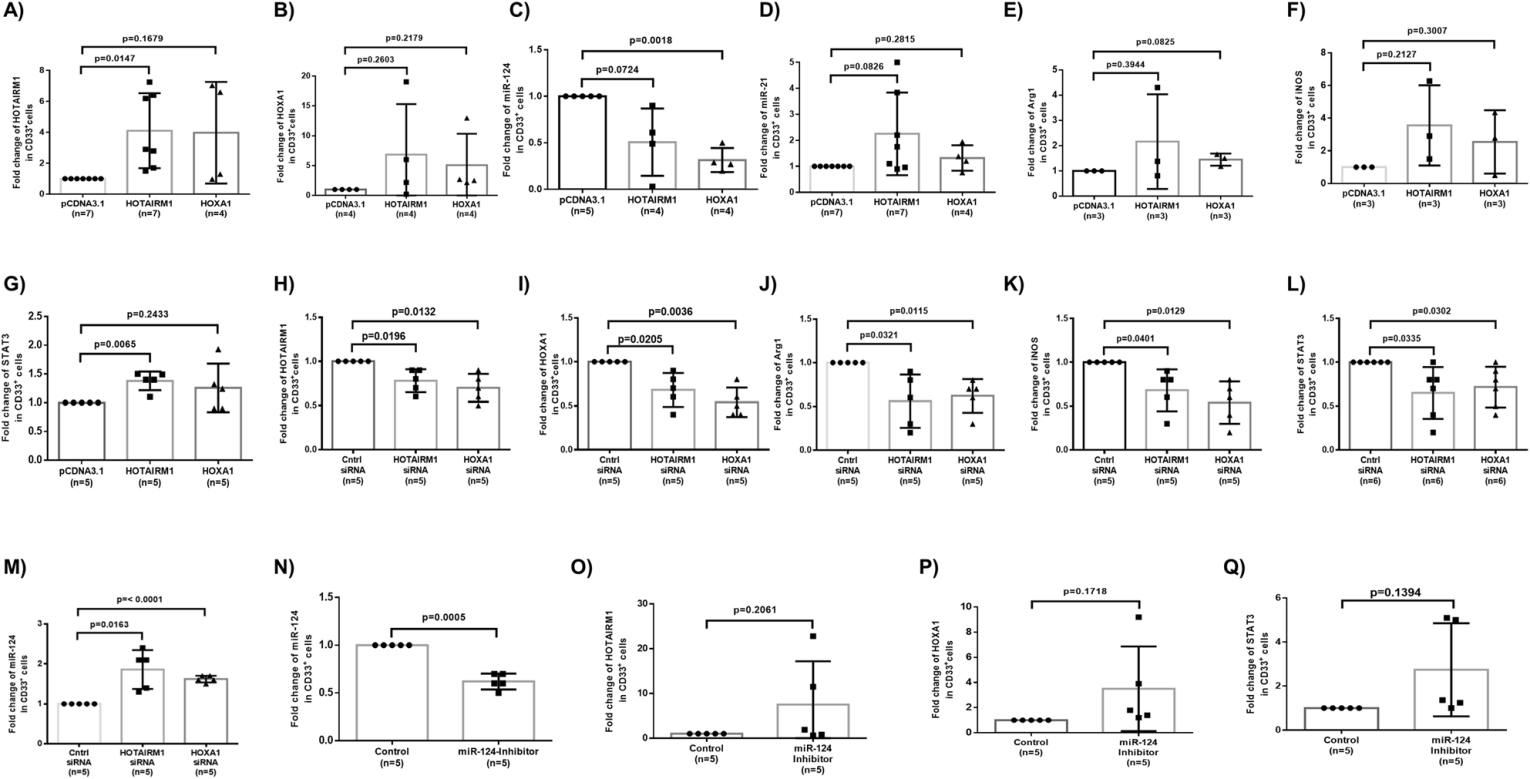
HOTAIRM1 and HOXA1 regulate each other and control miR124 expression in MDSCs. (**A–B**) HOTAIRM1 or HOXA1 expression levels in CD33^+^ cells transfected by pcDNA3.1 control, HOTAIRM1, or HOXA1 plasmids. (**C–D**) miR124 or miR21 expression levels in CD33^+^ cells transfected by pcDNA3.1 control, HOTAIRM1, or HOXA1 plasmids. (**E–G**) Arg1, iNOS, or STAT3 expression levels in CD33^+^ cells transfected by pcDNA3.1 control, HOTAIRM1, or HOXA1 plasmids. (**H–I**) HOTAIRM1 and HOXA1 expression in HCV-derived CD33^+^ cells transfected with HOTAIRM1, HOXA1, or control siRNA. (**J–M**) Arg1, iNOS, STAT3, and miR124 expression levels in HCV-CD33^+^ cells transfected with HOTAIRM1, HOXA1, or control siRNA. (**N–Q**) Expression levels miR124, HOTAIRM1, HOXA1, and STAT3 in HCV-CD33^+^ cells transfected with control or miR124 inhibitor.

To elucidate the cause-effect interrelationships between HOTAIRM1-HOXA1 and miR124/miR21 expressions, we transfected HS-derived CD33^+^ cells with HOTAIRM1 or HOXA1 expressing plasmids, followed by measuring their levels and miRNA expressions. As shown in Fig. 4A, HOTAIRM1 levels were upregulated (~ fourfold) in myeloid cells 5 days after HOTAIRM1 transfection. Interestingly, HOTAIRM1 expression was also upregulated (~ fourfold) by HOXA1 transfection, suggesting that HOXA1 may function as a transcription factor that positively regulates HOTAIRM1 expression. While transfection of HOXA1 upregulated (~ fivefold) HOXA1 levels, ectopic expression of HOTAIRM1 upregulated (~ sevenfold) HOXA1 levels (Fig. 4B), indicating that HOTAIRM1 enhances HOXA1 expression. Importantly, overexpression of either HOTAIRM1 or HOXA1 moderately downregulated miR124 levels (Fig. 4C), but only slightly upregulated miR21 expression (Fig. 4D). Overexpression of HOTAIRM1 or HOXA1 also slightly upregulated Arg1, iNOS, and STAT3 expressions (Fig. 4E-G).

In contrast to their overexpression, silencing of HOTAIRM1 and HOXA1 gene expressions by specific siRNAs transfection into CD33^+^ cells from HCV patients significantly reduced the levels of HOTAIRM1 and HOXA1 (Fig. 4H-I), further verifying the positive correlation between HOTAIRM1 and HOXA1. Silencing of HOTAIRM1 and HOXA1 also significantly downregulated Arg1, iNOS, and STAT3 levels, but upregulated miR124 level in HCV-CD33^+^ cells, which was decreased during chronic HCV infection (Fig. 4J–M), indicating the importance of HOTAIRM1/HOXA1 in controlling MDSCs’ suppressive functions. These results further demonstrate that HOTAIRM1/HOXA1 regulate the expression of immunosuppressive mediators/molecules in MDSCs during HCV infection.

Given that HOTAIRM1/HOXA1 expression appears to regulate miR124 level, we transfected CD33^+^ cells from HS with miR124 inhibitor and then examined HOTAIRM1/HOXA1 expressions. As shown in Fig. 4N, miR124 expression was significantly inhibited by the transfection of CD33^+^ cells with miR124 inhibitor. Notably, HOTAIRM1 (~ fivefold) and HOXA1 (~ 3.5-fold) expressions in CD33^+^ cells were upregulated by a miR124 inhibitor (Fig. 4O–P); correspondingly, the STAT3 level was upregulated (~ 2.5-fold) by transfection of CD33^+^ cells with the miR124 inhibitor (Fig. 4Q). In essence, our results suggest that HOTAIRM1 and HOXA1 positively regulates each other to control miR124 expression and enhance the immunosuppressive mediators in MDSCs, whereas miR124 may also feedback regulate HOTAIRM1, HOXA1, and STAT3 expressions in these cells.

### Ectopic expression of HOTAIRM1 and HOXA1 promotes MDSC differentiation and suppressive functions

**Figure 5:**
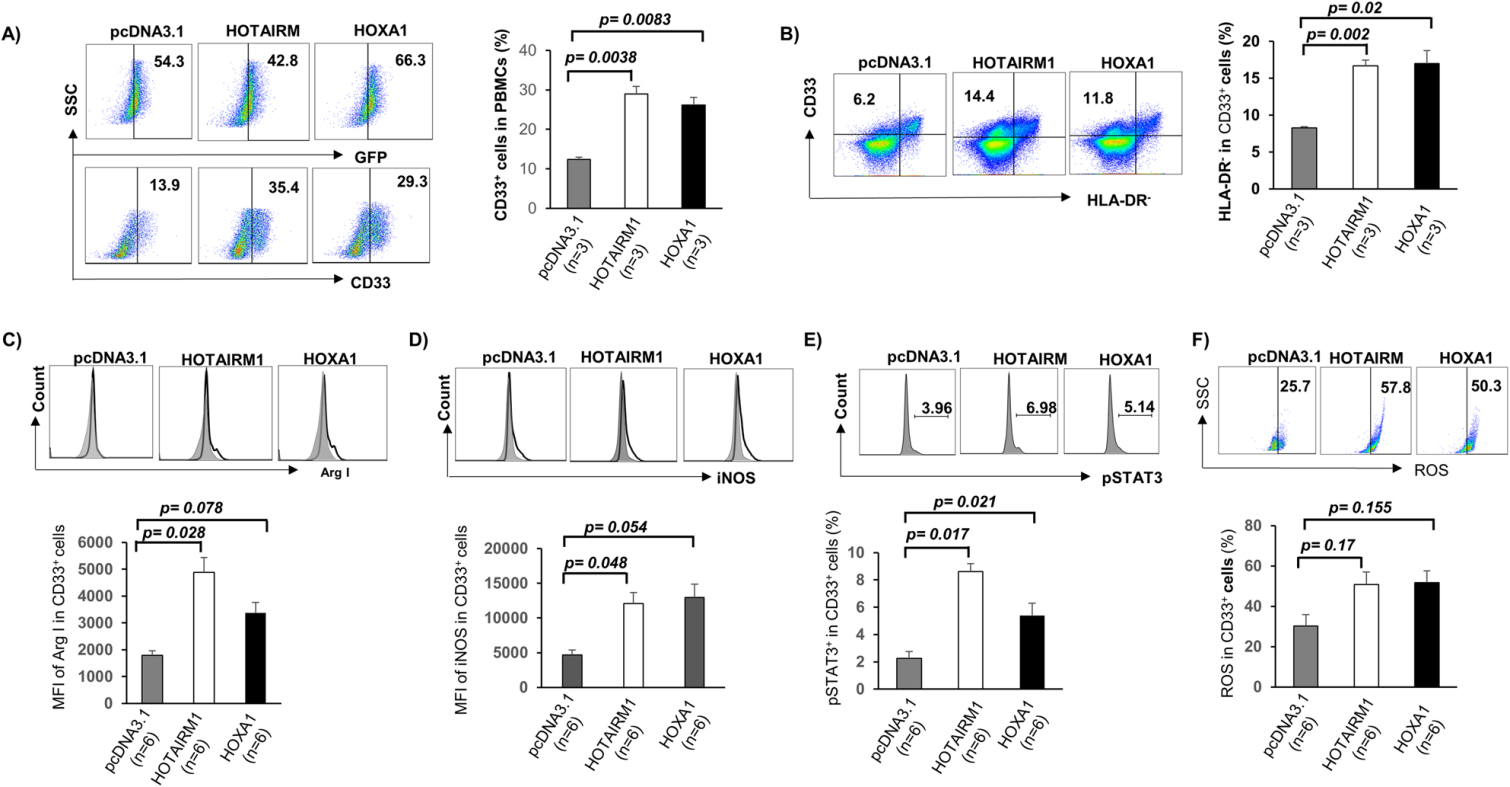
Ectopic overexpression of HOTAIRM1 or HOXA1 in healthy CD33^+^ cells affects MDSC differentiation and immunosuppressive functions. (**A**) Transfection of HOTAIRM1 or HOXA1 plasmid into PBMCs isolated from HS promotes CD33^+^ cell differentiation. (**B**) Ectopic expression of HOTAIRM1 or HOXA1 promotes CD33^+^ cell differentiation into MDSCs (determined by higher levels of HLA-DR^−^ cells). (**C–F**) Overexpression of HOTAIRM1 or HOXA1 in healthy CD33^+^ cells enhances Arg1, iNOS, pSTAT3, and ROS productions, as determined by flow cytometry.

Given the critical role of HOTAIRM1 and HOXA1 in myeloid cell differentiation, we hypothesized that ectopic expression of HOTAIRM1 or HOXA1 could induce MDSC development to suppress T cell functions. To test this hypothesis, we transfected healthy CD33^+^ cells with GFP-HOTAIRM1 or GFP-HOXA1 constructs or control plasmid (GFP-pCDNA3.1) for 3 days and then analyzed myeloid cell differentiation and the expression of immunosuppressive molecules. As shown in Fig. 5A, with transfection efficiency between 43%-66%, flow cytometry revealed that overexpression of HOTAIRM1 or HOXA1 resulted in a marked increase in myeloid cell differentiation. Compared to the empty vector control transfection, overexpression of HOTAIRM1 or HOXA1 in CD33^+^ myeloid cells resulted in an immunosuppressive phenotype, as evidenced by the higher levels of HLA-DR^−^ expression, a feature of MDSCs (Fig. 5B). Also, the expression levels of suppressive molecules, such as Arg1, iNOS, pSTAT3, and ROS were upregulated in the differentiated cells (Fig. 5C–F). These results suggest that the HOTAIRM1-HOXA1 axis promotes MDSC differentiation and suppressive functions.

### Silencing of HOTAIRM1 and HOXA1 expressions reduce MDSC frequencies and suppressive functions

**Figure 6:**
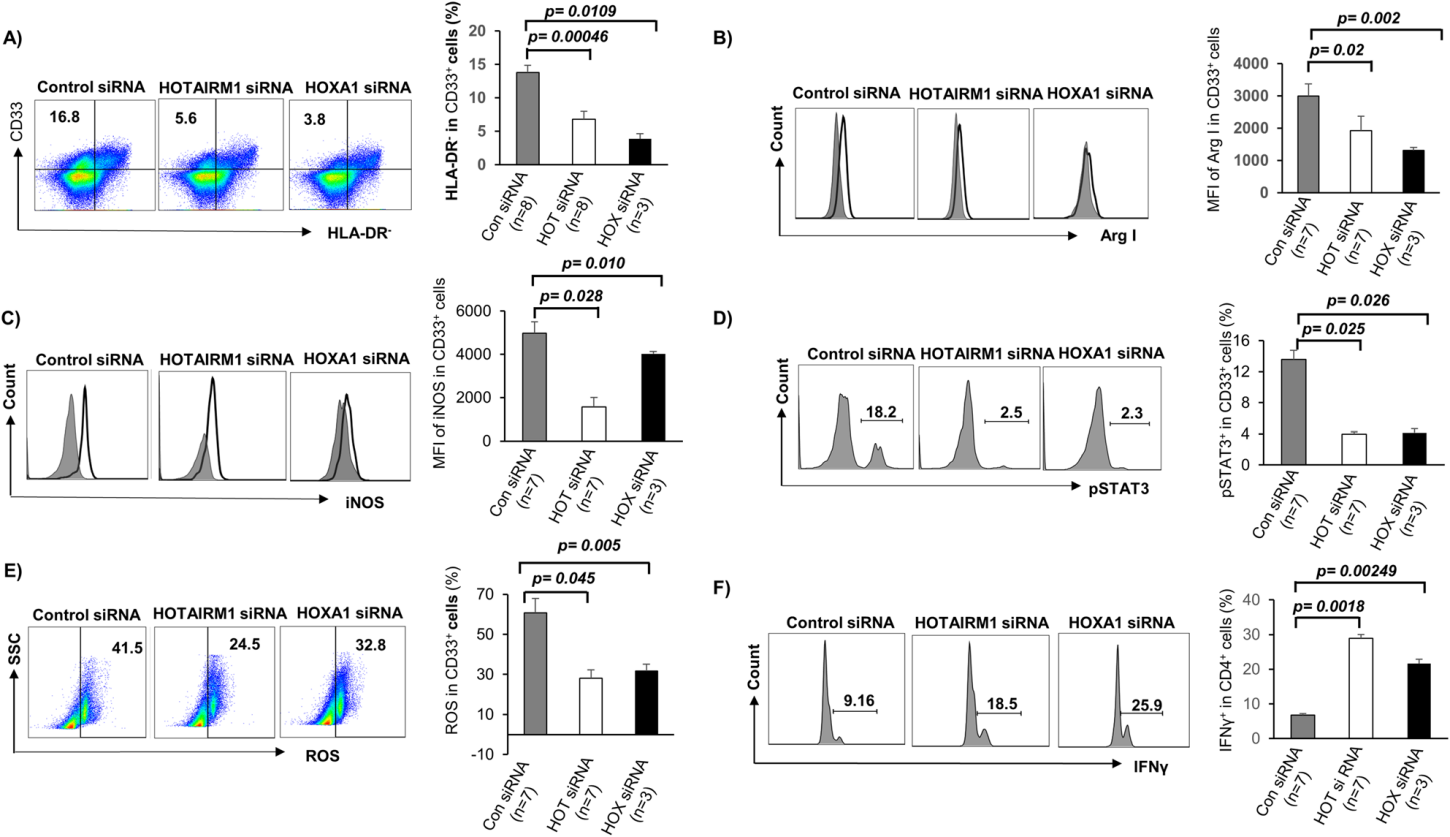
Silencing of HOTAIRM1 or HOXA1 expression in CD33^+^ cells from HCV-infected subjects abrogates MDSC differentiation and immunosuppressive functions. (**A**) siRNA-mediated knockdown of HOTAIRM1 or HOXA1 expression in CD33^+^ cells of HCV subjects inhibits CD33^+^ cell differentiation into MDSCs (determined by lower HLA-DR^−^ cell frequency). (**B–E**) siRNA-mediated knockdown of HOTAIRM1 or HOXA1 expression in CD33^+^ cells of HCV subjects inhibits Arg1, iNOS, pSTAT3, and ROS productions. (**F**) Improved IFN-γ production by HCV CD4 T cells following incubation with autologous CD33^+^ cells with HOTAIRM1 or HOXA1 knockdown.

We next asked whether silencing HOTAIRM1 or HOXA1expression can attenuate the HCV-induced MDSC expansion and immunosuppression. To this end, we transfected CD33^+^ cells derived from HCV subjects with HOTAIRM1 or HOXA1 siRNA. Compared to the control, HOTAIRM1 and HOXA1 siRNA significantly reduced the frequencies of CD33^+^ HLA-DR^−^ MDSCs (Fig. 6A). Transfection of these siRNAs also reduced the levels of immunosuppressive molecules Arg1, iNOS, pSTAT3, and ROS (Fig. 6B–E). Importantly, IFN-γ production by autologous CD4 T cells was restored when they were cultured with CD33^+^ cells with HOTAIRM1 or HOXA1 silencing (Fig. 6F). Taken together, these results indicate that MDSC differentiation and suppressive functions can be attenuated by inhibiting the HOTAIRM1-HOXA1 pathway.

### Targeting the HOTAIRM1-HOXA1 axis in MDSCs affects the T cell functions

**Figure 7:**
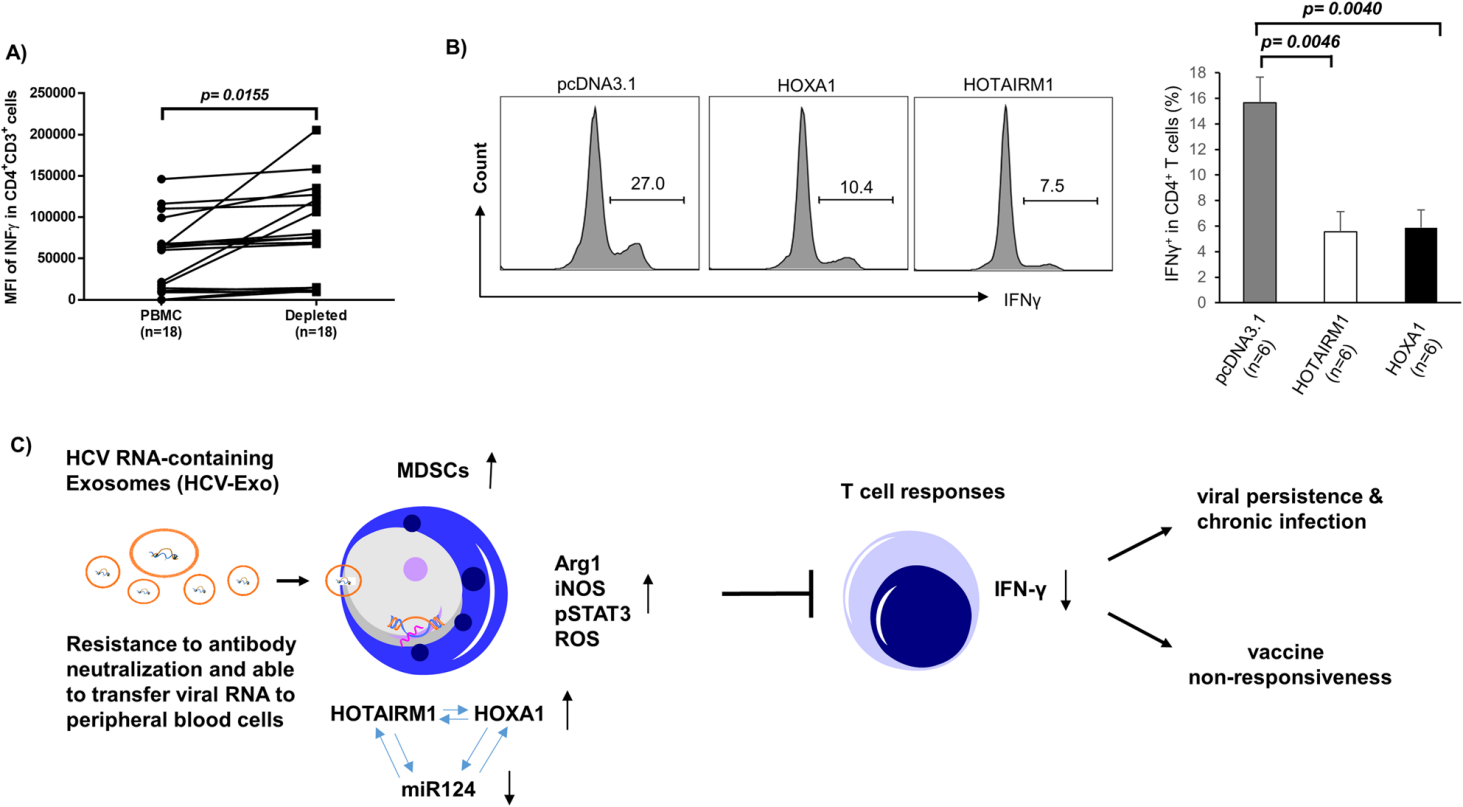
MDSCs suppress CD4 T cell functions. (**A**) Depletion of CD33^+^ cells from PBMCs of HS enhances IFN-γ production by CD4 T cells. (**B**) IFN-γ production is suppressed in CD4 T cells following incubation with autologous HS CD33^+^ cells overexpressing HOTAIRM1 or HOXA1. (**C**) A model describing the role of HOTAIRM1 in MDSC development and the mechanism by which it suppresses host immune responses. HCV RNA-containing exosomes (HCV-Exo) can induce MDSC differentiation and production of immunosuppressive molecules, such as Arg1, iNOS, pSTAT3, and ROS via induction of the HOTAIRM1-HOXA1-miR124 axis. HOTAIRM1 and HOXA1 can regulate each other's expression to decrease miR-124 levels and promote MDSC development, which in turn suppresses T cell functions, thus potentially contributing to viral persistence and vaccine non-responsiveness during chronic HCV infection.

To further consolidate the finding of MDSCs on suppressing T cell responses, we depleted CD33^+^ cells from PBMCs derived from HCV subjects. As shown in Fig. 7A, depletion of CD33^+^ cells from HCV PBMCs significantly increased the production of INF-γ in CD3^+^CD4^+^ T cells. This is consistent with our previous observations that IFN-γ production in CD4 T cells was enhanced by depleting CD33^+^ cells from PBMCs derived from virally infected subjects^13–16^. To further reveal the role of the HOTAIRM1-HOXA1 axis in regulating MDSC suppressive function, we overexpressed HOTAIRM1 or HOXA1 in healthy CD33^+^ cells, and then co-cultured them with autologous CD4 T cells for 3 days, followed by measurement of IFN-γ production in activated CD4 T cells. As shown in Fig. 7B, IFN-γ production was significantly suppressed in CD4 T cells co-cultured with CD33^+^ cells expressing HOTAIRM1 or HOXA1. These results support a role of HOTAIRM1-HOXA1 in promoting the MDSC immunosuppressive effect on T cell functions.

## Discussion

MDSCs have been shown to expand and inhibit host immunity in multiple disease models^9–16^; however, the mechanisms that regulate MDSC development during viral infection remain unclear. In this study, we demonstrated that the expression of lncRNA HOTAIRM1 and its target, the HOXA1 gene, are upregulated in MDSCs that accumulate during chronic HCV infection. Notably, the upregulation of HOTAIRM1 is closely associated with the expression of immunosuppressive molecules in MDSCs. Interestingly, HOTAIRM1 upregulation is induced by HCV-Exo, which can also downregulate miR124, and HOTAIRM1 or HOXA1 and can control miR124 expression in myeloid cells. Importantly, overexpression of HOTAIRM1 or HOXA1 in healthy CD33^+^ myeloid cells promotes MDSC differentiation and immunosuppressive functions. In contrast, silencing their expressions in MDSCs derived from HCV-infected subjects attenuates immunosuppressive functions. Based on these findings and our previous studies^13–16^, we propose a model (Fig. 7C) illustrating the role and mechanisms of HOTAIRM1 in promoting MDSC development to suppress the host immune response during HCV infection. According to this model, HCV-Exo can induce MDSC differentiation and their production of immunosuppressive molecules, such as Arg1, iNOS, pSTAT3, and ROS via promoting the HOTAIRM1-HOXA1-miR124 axis. HOTAIRM1 and HOXA1 can regulate each other in a positive loop to control miR124 expression and MDSC development, which in turn, suppresses T cell functions, thereby potentially contributing to viral persistence and vaccine non-responsiveness during chronic HCV infection.

Although our results show that HOTAIRM1/HOXA1 is upregulated and promote MDSC development during HCV infection, the underlying mechanisms remain to be determined. To date, while thousands of lncRNAs have been identified in humans, the roles and mechanisms of these lncRNAs in control of gene transcription and disease progression remain largely unknown. lncRNAs are key regulators of chromatin structure, affecting the epigenetic state and expression level of target genes through interactions with histone modifiers, chromatin remodeling complexes, transcriptional regulators, or the DNA methylation machinery^17–19^. In the nucleus, lncRNAs can act as a scaffold, recruiting activators or suppressors at target gene promoters, and can epigenetically regulate gene transcription by inducing histone modifications and chromatin remodeling. In the cytoplasm, lncRNAs can act as a sponge for miRNAs, which modify gene expression at the post-transcriptional level^49,50^. Here, we find that the lncRNA HOTAIRM1 can regulate HOXA1 as well as miR124 expression, and thus controls MDSCs development. These new findings further support our previous studies, which linked MDSC expansion to the induction of the STAT3 pathway by miR124^15^.

Our results suggest that HOXA1 is a target for positive regulation by HOTAIRM1. HOXA1, a HOXA gene cluster member, has been shown to be upregulated in human malignancies and acts as an oncogene. In our study, the pattern of HOTAIRM1 expression is rather similar to that of the HOXA gene, lending support to the notion that the intergenic non-coding transcription of the HOX genomic regions is crucial to maintaining the active state of HOX clusters. Notably, HOX clusters have a specific pattern of lineage-restricted expression, where HOXA genes are predominantly expressed in myeloid cells^51^. The upregulation of some genes of the HOXA cluster has been observed in several subtypes of acute myeloid leukemia (AML)^52,53^. Mechanistically, HOTAIRM1 contributes to three-dimensional chromatin organization changes that are required for the temporal collinear activation of HOXA genes^30^. HOTAIRM1 also contributes to the physical dissociation of chromatin loops at the cluster proximal end, which delays recruitment of the histone demethylase UTX and transcription of central HOXA genes^30^. In addition, a previous study reported that HOTAIRM1 mediates demethylation of histone proteins and reduces DNA methylation levels via epigenetic modulation of HOXA1 gene expression^29^. This finding provides an example of transcriptional control via the chromatin state and may help explain the role of HOTAIRM1 within the HOXA gene cluster. Thus far, how HOTAIRM1 and HOXA1 control each other's expression in a mutually exclusive manner is unclear. DNA sequence analysis of HOTAIRM1 shows a structure with the presence of a bi-directional promoter shared by the divergent coding and noncoding RNAs that may facilitate the *cis* action of HOTAIRM1 on their regulating genes^19,20^. Notably, HOTAIRM1 and HOXA1 are not always coordinately expressed, indicating that HOTAIRM1 may also be transcribed independently^21^. These studies indicate a positive feedback loop in regulation of the HOXA1 and HOTAIRM1 to control the MDSCs differentiation and suppressive function.

Interactions between lncRNAs, miRNAs, and mRNAs have been described previously, which show a multilayered complexity of RNA crosstalk and competition^54,55^, and that lncRNAs seem to regulate both the expression of neighboring genes and distinct genomic sequences^56^. Interestingly, the HOX genomic regions have numerous ncRNAs, suggesting that these ncRNAs may participate in the regulation of HOX expression^21^. Specifically, HOTAIRM1 can regulate HOXA1 and HOXA4 expressions^24^. Also, HOTAIRM1 regulates myeloid maturation in human NB4 promyelocytic leukemia cells^27^. Our current study clearly shows that HOTAIRM1 and HOXA1 can regulate each other to control miR124 levels in MDSCs. This is in line with a recent report showing that the HOTAIR-miR214 axis plays an important role in the proliferation, migration, and invasion of hepatocellular carcinoma^57^. The cooperation of two ncRNAs, HOTAIRM1 and miR124, in the MDSC development and disease progression merits further investigation.

While many studies are investigating lncRNAs as potential biomarkers and therapeutic targets for human diseases, to our knowledge, this is the first report showing that the HOTAIRM1-HOXA1-miR124 axis promotes MDSCs development and immunosuppressive functions during chronic HCV infection. Therefore, targeting this axis may provide a novel approach for immunotherapy in conjunction with antiviral therapy to combat human viral diseases.
